# Co-Evolution of Social Learning and Evolutionary Preparedness in Dangerous Environments

**DOI:** 10.1371/journal.pone.0160245

**Published:** 2016-08-03

**Authors:** Björn Lindström, Ida Selbing, Andreas Olsson

**Affiliations:** 1 Department of Clinical Neuroscience, Karolinska Institutet, Stockholm, Sweden; 2 Department of Economics, University of Zurich, Zürich, Switzerland; Centre for Coevolution of Biology & Culture, University of Durham, UNITED KINGDOM

## Abstract

Danger is a fundamental aspect of the lives of most animals. Adaptive behavior therefore requires avoiding actions, objects, and environments associated with danger. Previous research has shown that humans and non-human animals can avoid such dangers through two types of behavioral adaptions, (i) genetic preparedness to avoid certain stimuli or actions, and (ii) social learning. These adaptive mechanisms reduce the fitness costs associated with danger but still allow flexible behavior. Despite the empirical prevalence and importance of both these mechanisms, it is unclear when they evolve and how they interact. We used evolutionary agent-based simulations, incorporating empirically based learning mechanisms, to clarify if preparedness and social learning typically both evolve in dangerous environments, and if these mechanisms generally interact synergistically or antagonistically. Our simulations showed that preparedness and social learning often co-evolve because they provide complimentary benefits: genetic preparedness reduced foraging efficiency, but resulted in a higher rate of survival in dangerous environments, while social learning generally came to dominate the population, especially when the environment was stochastic. However, even in this case, genetic preparedness reliably evolved. Broadly, our results indicate that the relationship between preparedness and social learning is important as it can result in trade-offs between behavioral flexibility and safety, which can lead to seemingly suboptimal behavior if the evolutionary environment of the organism is not taken into account.

## 1. Introduction

Danger is a fundamental aspect of the lives of most animals. A key dimension of adaptive behavior is therefore to avoid actions, objects, and environments associated with danger. However, because animals also must forage for food and mates, adaptive behavior involves balancing avoidance of danger and approach of possible rewards [1]. Predation is the foremost danger in the natural world, and is believed to exert a strong selective pressure across evolutionary time, for both humans [2] and non-human animals [3].

The selective pressure posed by such dangers drives the evolution of adaptations. Anti-predator adaptations include morphological defenses, such as crypsis, and behavioral defenses, including group-living, vigilance and avoidance behaviors [2,4]. However, if the environment is temporally or spatially heterogenic, genetically rigid behavior programs with hardwired input-output relations might not be adaptive. Instead, phenotypic or behavioral plasticity will be crucial for survival [5,6]. Learning allow animals to adapt their behavior to the statistic regularities of the environment, thereby associating certain stimuli or actions with rewards, such as food or sexual opportunities, and others with danger, such as predators, noxious objects or threatening con-specifics. Behavioral mechanisms of phenotypic plasticity might however also have fitness costs; if the environment is dangerous, individual trial-and-error learning can expose the animal to high risks and costs [7]. For example, foraging for novel food can expose an animal to risks (e.g., poison) that are avoided by animals with genetically specified and rigid food preferences. Adaptations that reduce such costs are therefore predicted.

In the present study, we investigated the interaction of two such cost-reducing behavioral adaptions through evolutionary agent-based modeling, (i) genetic coding of preferences (*preparedness*) and (ii) social learning. The existence of both types of adaptations is empirically well-established, but when and how they can be expected to interact is currently unclear. As we will describe in the following sections, there are empirical reasons to believe that the interaction of preparedness and social learning can be both synergistic and antagonistic, in humans as well as non-human animals. Our goal in the present study was to understand when and why each tendency (synergistic or antagonistic interaction) might evolve and be expressed, by simulating how agents behave in a dangerous decision making environment based on empirically well-grounded learning mechanisms [8,9]. We believe the interaction of preparedness and social learning could be of importance for non-human animals (e.g., primates) that are reliant on social learning, and in particular for humans where the use of social learning is of fundamental importance for adaptive behavior [10].

Biological constraints on learning (*preparedness*) serve to promote approach of fitness-beneficial stimuli and avoidance of dangerous stimuli [11–14]. That learning, in the form of both classical and instrumental conditioning, is biologically constrained and reflect species-specific evolutionary contingencies [7] has been acknowledged since at least the 70s [15,16]. In humans and other primates, a range of danger-related stimuli, primarily exemplified by snakes and threatening con-specific faces, are more easily and persistently associated with fear through learning than neutral control stimuli [16–18]. Such preferential learning was taken as evidence for preparedness, a quantitative dimension ranging from prepared to contra-prepared [15]. Preparedness is thought to underlie the asymmetric distribution of specific phobias in humans; snake and spider phobias are over-represented while stimuli that pose real danger to modern day humans in the western world, such as cars or electric outlets, seldom are object of phobias [15,16]. While limited experimental control of the individuals’ learning history preclude strong conclusions about innate predispositions in humans, it is known that a range of non-human animal species exhibit innate behavioral responses to species-typical predators [19–21]. Importantly, such innate responses can be potentiated by learning, suggesting that they are related, or analogue, to preparedness as identified in experimental psychology [22]. Presumably, genetic preparedness will evolve toward stimuli that pose recurring threats or opportunities on an evolutionary timescale, especially if the first interactions with the stimulus can be fatal [7]. In support of this, experiments using experimental evolution have shown that animals (*Drosophila* flies) can acquire genetic, innate tendencies for both avoidance of an aversive, fitness-detrimental stimulus [23,24] and approach of a reward-related fitness-beneficial stimulus [25] in less than 40 generations, highlighting the fact that preparedness might evolve rapidly given sufficient selective pressure. Together, such findings suggest that many species likely have some degree of preparedness toward predators or other objects associated with danger, and that preparedness is not generally expressed in rigid behavioral programs [23]. Rather, preparedness facilitates adaptive behavior together with learning by providing an “evolutionary prior” upon which preferences are constructed [26].

Social learning represents a radically different type of behavioral adaptation that can reduce the costs of individual trial and error foraging [27]. Exploiting the knowledge of others through social learning allows the individual to avoid exposure to the risks associated with individual learning. This function of social learning was originally recognized by Boyd and Richardson (1995), who showed with population genetic modeling that social learning is adaptive when information is costly to acquire individually (“costly information hypothesis”). Laland (2004) rephrased this function in an influential taxonomy of social learning strategies as “copy when asocial learning is costly” (henceforth “copy when costly”) [28]. Many animal species learn about predators through social means [29]. A well-known example is the acquisition of snake fear among snake-naïve captive rhesus monkeys who observed con-specifics behaving fearfully toward (toy) snakes [30,31]. Similarly, black birds acquire fear response of predators through association with the alarm calls of a con-specific [32]. Further complicating the understanding of social learning in dangerous environments is the important distinction concerning the direction of the social influence; horizontal (within the same generation) or vertical (between generations). Presently, the majority of the studies supporting the “copy when costly” strategy has focused on horizontal social transmission. However, much evidence suggests that vertical transmission is important for how humans learn about the dangers and opportunities in the environment. For example, among hunter-gatherers in the Congo basin, social learning is primarily vertical under age 5 and horizontal (or oblique) between the ages of 6 and 12 [33]. Parental influences on offspring, either as models for observational conditioning [34] or direct verbal or nonverbal teaching [35], is thought to be an important route for fear and avoidance learning [36]. This distinction between horizontal and vertical transmission has been relatively overlooked in previous discussions regarding how social learning allows avoidance of costly dangers, but is likely to be important for understanding how preparedness and social learning coevolve, wherefore our model included social learning strategies corresponding to both horizontal and vertical transmission (see section 2. Model description).

Important, but not commonly acknowledged, is that preparedness can influence social learning as well as individual learning. In humans, the signatures of prepared learning have been shown in observational conditioning to snake stimuli [37], a finding mirrored by how Rhesus monkeys acquire fear of snakes, but not flowers, through observing the fearful responses of con-specifics [30,31]. Similarly, blackbirds acquire a stronger fear response toward a model bird of an unfamiliar species than to control stimuli (a plastic bottle), following observational conditioning [32]. Recent studies with human children have shown enhanced social learning about dangerous animals cross-culturally [38,39]. Despite the fact that these two types of cost reducing adaptations, preparedness and social learning, are likely to interact if the environment is dangerous, no studies have investigated how this interaction shape the evolution of adaptive behavior. The examples above indicates that the effect of preparedness on social learning is a synergistic, but given the similarities between asocial and social learning [40,41], there are reasons to predict antagonistic interactions as well. There are many examples of adverse effects of preparedness on behavior in humans and non-human animals [42–45], with the common underlying theme that preparedness can lead to inflexible behavior, which might be detrimental if the environment is changing.

In the present study, we asked three inter-related questions about the two cost reducing adaptions—genetic preparedness and social learning—reviewed above. Given the wide evidence for both the existence of preparedness mechanisms across species, and the importance of social learning in avoidance of danger, especially for humans, it is pertinent to analyze how, and when, these mechanisms evolve, and how they interact. Specifically, we asked (i) if preparedness and social learning typically both evolve in dangerous environments, (ii) if these adaptions typically interact synergistically or antagonistically, and (iii) what type of environmental factors lead to synergistic and antagonistic interactions, respectively.

To address these questions, we used agent-based evolutionary simulations that explored the evolutionary interactions of preparedness and social learning in dangerous and stochastic environments. Individual agents learned to associate different choice options (e.g., stimuli or patches) with reward (e.g., food) through experience in a multi-armed bandit environment [46]. Some of the available choice options could result in fatal outcomes. We explored how the level of danger of the environment (i.e., the number of possibly fatal choice options) together with reward stochasticity affected the success of individual learning and three types of social learning strategies: asocial learning combined with observational learning (horizontal transmission), asocial learning combined with parental learning (vertical transmission), or the combination of asocial learning, observational learning and parental learning (horizontal and vertical transmission).

The evolution and behavior of these learning strategies was compared in the presence, relative to the absence, of preparedness to answer the questions posed above. We considered two distinct indices of adaptive behavior: foraging efficiency, and risk of death (under which we subsume any fatal interaction with a dangerous stimulus or action) [47]. The stochasticity of the environment was varied so that the environment either was stationary, slowly changing (between generations), or rapidly changing (within generations), a distinction previously shown to be important for the adaptiveness of learning per se [48], and the evolution of social learning [49,50]. To provide an unbiased account of the interaction of preparedness and social learning, we neither assumed any intrinsic costs nor benefits to the use of social learning nor were the agents restricted to using either asocial or social learning. Thus, any benefits or costs of both preparedness and social learning arose purely from the behavioral mechanism’s effect on individual behavior.

Agent-based modeling allowed us to directly and explicitly incorporate empirically supported learning rules into evolutionary dynamics. The behavior of each individual agent was controlled by a simple Rescorla-Wagner reinforcement learning (RL) algorithm [51]. Reinforcement learning, where the expected values of actions are updated based on the difference between experienced and expected reinforcement, can account for both behavioral [51] and neural aspects of both individual [52–54], and social learning [55–57]. There has been increasing recognition that behavioral mechanisms, such as learning rules, constrain the flexibility of adaptive behavior [8], and that evolutionary modeling therefore can benefit from explicitly incorporating such mechanisms [9].

It is important to note that the computational or cognitive mechanisms underpinning social learning are debated [40,41]. This debate centers on the question whether social learning simply is the consequence of applying the same learning mechanisms that govern individual learning to social stimuli, or a dedicated cognitive system specialized for social learning [40]. In this paper, when referring to social learning as an adaption under selection, we mean that learning from others can be beneficial in certain environments, and not that social learning is a specialized cognitive system under selection. The computational implementation of social learning in our model is identical to individual learning, and thereby compatible with social learning as arising from preferential attention to social stimuli rather than dedicated cognitive mechanisms [41]. Social learning can thereby be seen as an exaptation, which uses pre-existing learning mechanisms, evolved for individual learning.

## 2. Model Description

### 2.1 The environment

The environment consists of a 10-armed “bandit”, where the ten arms can be interpreted as representing different stimuli (i.e., food or a patch) or actions. The multi-armed bandit framework has previously been used to analyze the conditions favoring social learning [46].

The reinforcement, *R*, associated with each arm, henceforth referred to as option, was a positive integer, randomly drawn from the range [0,20] at the start of the simulation. The environment was temporally stochastic: with probability *P*_*change*_ the reward of one randomly selected option was randomized anew from [0,20] on each time-step.

The number of dangerous option, i.e. the number of option that have an increased death Probability [0 < *P*_*danger*_ <1], is denoted by *Danger* and we consider the consequences of Danger = 0,1,…,7 on the evolution of preparedness and social learning. Together, these two parameters (*P*_*danger*_ and *Danger*) determined how dangerous the environment was. Importantly, because choosing a dangerous option only probabilistically (*P*_*danger*_) resulted in a fatal outcome, the dangerous options could be associated with reward through both individual and social learning. Following preliminary simulations, we focused on the *Danger* parameter, which had the strongest influence on the evolutionary dynamics, and fixed *P*_*danger*_ to 0.5. For the main analyses, the same options were dangerous through the simulation, which corresponds to environments where the same predators or food-types constitute recurrent threats across evolutionary time.

### 2.2 The population

We simulated a population of *n* = 100 haploid asexually reproducing agents. Genetic preparedness (*P*) was regulated by 10 quantitative loci (*P*_*1*_,..,*P*_*10*_*)*. At the start of the stimulations, *P*_*1*,..,*10*_ = 0. Social learning (observational conditioning and parental learning) was regulated by two loci, each with two alleles (Observational conditioning: *O*_*0*_, *O*_*1*._ Parental learning: *T*_*0*_, *T*_*1*_), resulting in four learning strategies: asocial learning only (*O*_*0*_ + *T*_*0*_), observational learning (*O*_*1*_ + *T*_*0*_), parental learning (*O*_*0*_ + *T*_*1*_), and advanced social learning (*O*_*1*_ + *T*_*1*_). At the outset of the simulation, all agents were asocial learners (*O*_*0*_ + *T*_*0*_).

At the end of each time-step the surviving agents reproduced until the fixed population size (n = 100) was reached (see section 2.5). The reproduction probability (*P*_*Reproduce*_) was based on *fitness*, the cumulative reward (see Eq 4) received until time step *T*

$$f_{i}=\Sigma_{t=1}^{T}R\left(t\right)$$ (1)

$$P_{Reproduce,i}=\frac{f_{i}\;}{\begin{array}{c} \text{max}\\ j\in \left\{1,..,n\right\} \end{array}f_{j}}$$ (2)

where *f*_*i*_ refer to the cumulative reinforcement (*R*) [58] of individual *i* at time-step *T*, and the denominator is the maximum cumulative reinforcement in the population at the same time-step, resulting in a probabilistic ordering of the population according to foraging efficiency (i.e., fitness). Each individual could reproduce multiple times at each time-step. Note also that if no agents died at a given time-step, no reproduction took place (so a high *P*_*Reproduce*_ does not necessarily result in reproduction).

The agents introduced by reproduction inherited the genetic preparedness and learning strategies of the parent. At reproduction, one mutation was introduced with probability 0.03. The locus of this mutation was random, resulting in a mutation probability of 0.0025 for each of the twelve loci (*P*_*1*_,…, *P*_*10*_, *O*,*T*). Each *P* trait could be modified by mutation: *P(t+1) = P(t)* + ε, ε ~ *U*(-20,20), i.e., a uniformly distributed random number with max value equal to the maximum reward in the environment. The learning strategy loci mutated bidirectional in a binary manner (e.g., *O*_*0*_ *→ O*_*1*,_ *O*_*1*_ *→ O*_*0*_). For simulations without genetic preparedness, mutations at the learning strategy loci had the same probability (0.0025) as for simulations with genetic preparedness.

### 2.3 Learning abilities

All agents could learn asocially. The asocial learning rule was a simple reinforcement learning (RL) algorithm, equivalent to the Rescorla-Wagner model. This rule is known to provide a comprehensive account of both human and non-human trial-and-error learning [51,54,59], and to be evolutionary robust [60]. The rule states that the expected value *Q*_*i*_(*t* + 1) of the option *i* at time-point *t* + 1 is the sum of the previous value of the action *Q*_*i*_(*t*) and the prediction error *δ*_*i*_(*t*) multiplied with the learning rate α (0 < α < 1. Set to 0.3 in all simulations):

$$Q_{i}\left(t+1\right)=\;Q_{i}\left(t\right)+\;\alpha \times \delta_{i}\left(t\right)$$ (3)

The prediction error, *δ*_*i*_(*t*), is the difference between the expected outcome value of the action, *Q*_*i*_(*t*), and its actual reinforcement value, *R*_*i*_(*t*):

$$\delta \left(t\right)=R_{i}\left(t\right)-Q_{i}\left(t\right)$$ (4)

At the start of the simulation, and when new agents were introduced by reproduction, the expected value of each option was set to the average reward of the environment (i.e., *R*_*i*_*(t = 1)* = 10).

Agents with the observational conditioning allele (*O*_*1*_) furthermore learned by observing a randomly selected individual in the population at each time-step (subsequent to choosing among the options themselves). Observational conditioning was identical to individual learning (Eqs 3 and 4): without error, the individual updated *Q*_*i*_ by the reward of the observed individual choosing option *i*, with the same learning rate, α, as in individual learning. If the observed individual received a (highly negative) fatal outcome, the reinforcement, *R*, used to update the expected value was set to -1000.

Agents with the parental learning (*T*_*1*_) transmitted the expected value, *Q*_*i*_, of all options *i* at the time of reproduction to their offspring. Because our impetus was to investigate the relationship between evolutionary preparedness and social learning, parental learning (as well as observational conditioning) was error-free and not associated with any intrinsic costs. Thus, the present implementation represent as a “best case scenario” for social learning.

### 2.4 Decision rule

The agents chose at each time-step among all options. The choice rule was an exponential ratio rule (commonly referred to as *Softmax*), which calculates the probability of choosing option *i* by weighing the expected value of *i* against the values of all others options [61] resulting in higher probabilities for options with high expected values:

$$Pr_{i}=\;\frac{e^{Q_{i}+\;P_{i}}}{\sum_{j=1}^{10}e^{Q_{j}+\;P_{j}}}$$ (5)

Here, the expected value of each option is the sum of the learned expected value of the option (*Q*_*i*_) and the preparedness associated with the option (*P*_*i*_), resulting in competition between the learned values and preparedness for behavioral control. This implementation allows preparedness to function as an “evolutionary prior” on the learned values by increasing the probability for actions with positive preparedness (*P* > 0) and decreasing the probability of actions associated with negative preparedness (*P* < 0) [26]. Thereby, this model leads to a non-uniform distribution of action probabilities in the absence of learning if any of the 10 preparedness traits in non-zero. It is important to note that this can result in both synergistic and antagonistic effects of preparedness on adaptive behavior, depending on how well the genetic preparedness corresponds to the rewards available in the current environment. Learning models similar to the present implementation has been successfully used to account for the interaction of preparedness and individual learning on human behavior [43].

### 2.5 Scheduling

The simulation proceeded in discrete time-steps. On each time-step, the following events occurred: (i) the environment changed with probability *P*_*change*_, (ii) each individual chose one option based on the learned values and evolutionary preparedness, (iii) agents with the observational conditioning allele, *O*_*1*_, updated the value of the option chosen by the observed individual based on that agents reward, (iv) agents that received a fatal outcome was removed, (v) the surviving agents updated the expected value of the option chosen (Eqs 3 and 4), (vi) *P*_*reproduce*_ was calculated (Eqs 1 and 2), (vii) agents died with probability 0.02, (vii) the agents probabilistically (based on *P*_*reproduce*_) reproduced in a random order until the original population size was replenished. All simulations were run for 50000 time steps, and all analyses conducted on the last 25000 time steps. See S1–S3 Codes for implantation of the model.

## 3. Results

**Fig 1:**
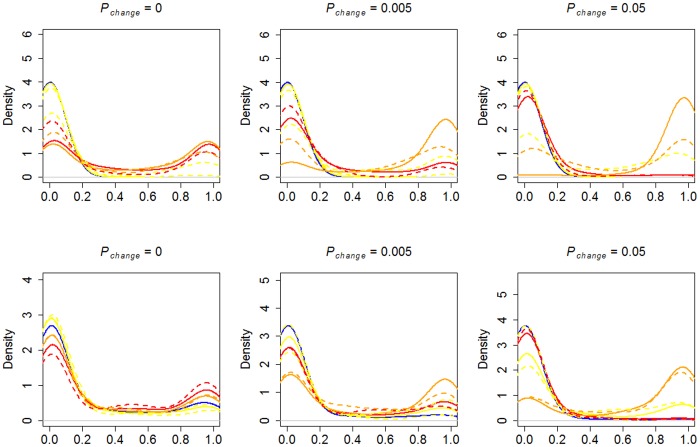
Phenotypic frequency densities without (top) and with (bottom) preparedness. Density plot for the phenotypic frequency at the end-point (time step 50000) for the different strategies over 100 simulation runs. Solid lines: *Danger* > 0. Dotted lines: *Danger* = 0. Blue = Asocial learning (*O*_*0*_,*T*_*0*_), yellow = observational learning (*O*_*1*_,*T*_*0*_), red = parental learning (*O*_*0*_,*T*_*1*_), orange = advanced social learning (*O*_*1*_,*T*_*1*_).

We first explored how preparedness affected the evolution of asocial and social learning in environments characterized by different degrees of danger and reinforcement stochasticity. All populations began as asocial learners. To provide a baseline for the effect of preparedness we show in Fig 1 (top) the phenotypic frequency densities of learning strategies in agents *without* preparedness.

**Fig 2:**
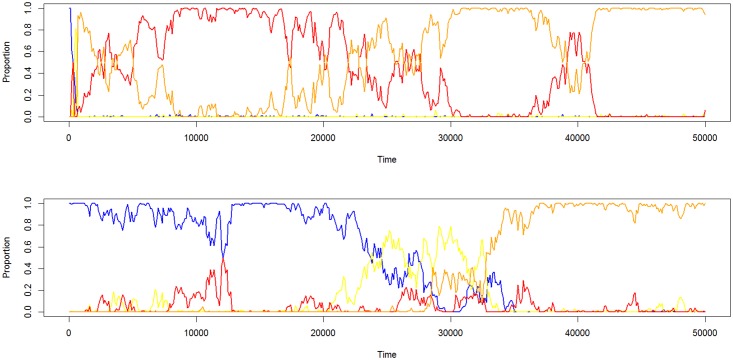
Example evolutionary dynamics without (top) and with (bottom) preparedness. Blue = Asocial learning (*O*_*0*_, *T*_*0*_), yellow = observational learning (*O*_*1*_,*T*_*0*_), red = parental learning (*O*_*0*_,*T*_*1*_), orange = advanced social learning (*O*_*1*_,*T*_*1*_). *Danger* = 7, *P*_*change*_ = 0.05.

In clear correspondence with the “copy when costly” strategy, dangerous environments (*Danger* > 0) led to selection for the advanced social learning phenotype (*O*_*1*_ + *T*_*1*_), but only when the environment was stochastic (*P*_*change*_ > 0). In stationary environments (*P*_*change*_ = 0), the advanced social learning phenotype and the parental learning phenotype (*O*_*o*_ + *T*_*1*_) were both relatively successful. This pattern indicates that vertical transmission is selected for in dangerous environments; intuitively, having a representation of dangerous actions at birth prevents costly errors [7]. However, rather than occupying a mixed equilibrium, these strategies tended to be dominant in separate simulation runs (see S1 Fig for the across run stochastic equilibrium distributions of each strategy, and S2 Fig for end point frequencies). Fig 2 shows examples of the evolutionary dynamics in a dangerous and stochastic environment in simulations without (top) and with (bottom) preparedness.

In Fig 1 (bottom), the average phenotypic frequency densities of learning strategies in agents *with* preparedness are shown. As evident, the inclusion of preparedness led to a different pattern of results. Most apparent, and particularly pronounced in stationary environments, selection for social learning strategies was weaker in the presence of preparedness than in its absence. In stationary environments, preparedness allows the agents to adapt to the environment without learning. In similarity to the simulations without preparedness, the advanced social learning strategy tended to be most successful in more stochastic environments.

Comparing the pattern in populations with (Fig 1, bottom) and without (Fig 1, top) preparedness, it is notable that the pure observational learning (*O*_*1*_ + *T*_*0*,_ yellow lines) is on average unsuccessful when the environment is dangerous (*Danger* > 0), and stationary or changing between generations (*P*_*change*_ = 0.005). This pattern indicate that the vertical social transmission allele (*T*_*1*_) has a very pronounced effect on foraging efficiency (see also Fig 2), which however was attenuated in the presence of preparedness. In turn, this attenuation indicates, in agreement with intuition, that preparedness and vertical social transmission can play similar roles, i.e., transmit preferences from parents to offspring.

It should also be noted that an increase in the rate of environmental change lead to a higher degree of success for the advanced social learning strategy, both in the absence and presence of preparedness. This pattern is consistent with previous models of social learning, which have shown that evolution tend to favor social learning if the environmental rate of change is intermediate [49,50].

### 3.1 The direction of preparedness

**Fig 3:**
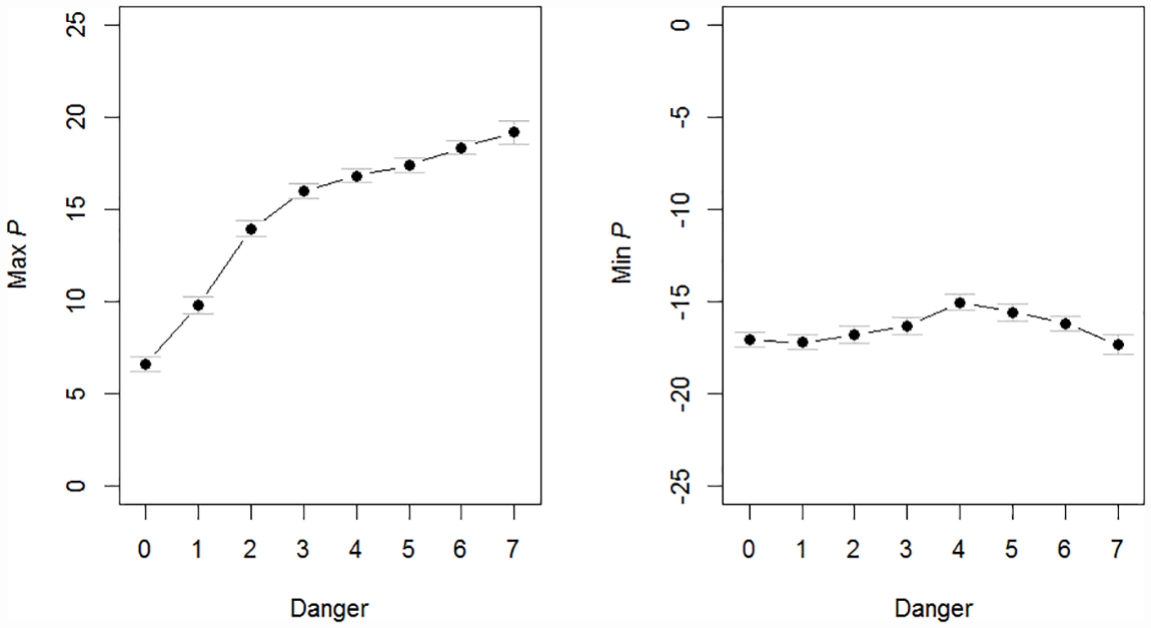
Preparedness as a function of environmental danger. Average max and minimum values of the preparedness vector regulating innate approach/avoidance tendencies averaged over all levels of *P*_*change*_. The means are derived from averaging time-step 25000–50000 for 100 repeats of each simulation. The standard errors are calculated across the 100 runs.

To further clarify the influence of preparedness on the evolution of social learning strategies in dangerous environments, we analyzed the preparedness (*P*) vector in populations with preparedness. Fig 3 shows the mean maximum and minimum of the *P* vector for different levels of *Danger* (this pattern was highly similar for the different levels of *P*_*change*_ and is therefore averaged across these for simplicity). Positive preparedness (*P* > 0, Fig 3, left), which is expressed as an innate approach tendency or preference for certain actions, increased nearly linearly with *Danger*, suggesting that in highly dangerous environments, it is critical for survival to focus actions on the few available safe options. This can be seen as an example of the “frame problem”; given the multitude of possible actions that can be taken, how do organisms limit their actions to fitness-promoting ones? [38]. Positive preparedness provides a solution to this problem by strongly increasing the probability of choosing the safe actions. At the highest levels of environmental danger, this might be a more efficient solution than genes promoting avoidance of all the other options. Negative preparedness however also contributes to adaptive behavior (Fig 3, right); the probability of choosing an option is proportional to the *difference* in value between actions (Eq 5), meaning that positive and negative preparedness interact to promote avoidance of the dangerous options. In contrast to positive preparedness, negative preparedness (*P* < 0, Fig 3, right) was similar across different levels of environmental danger, including environments without any dangers.

### 3.2 Preparedness leads to a trade-off between foraging efficiency and survival

**Fig 4:**
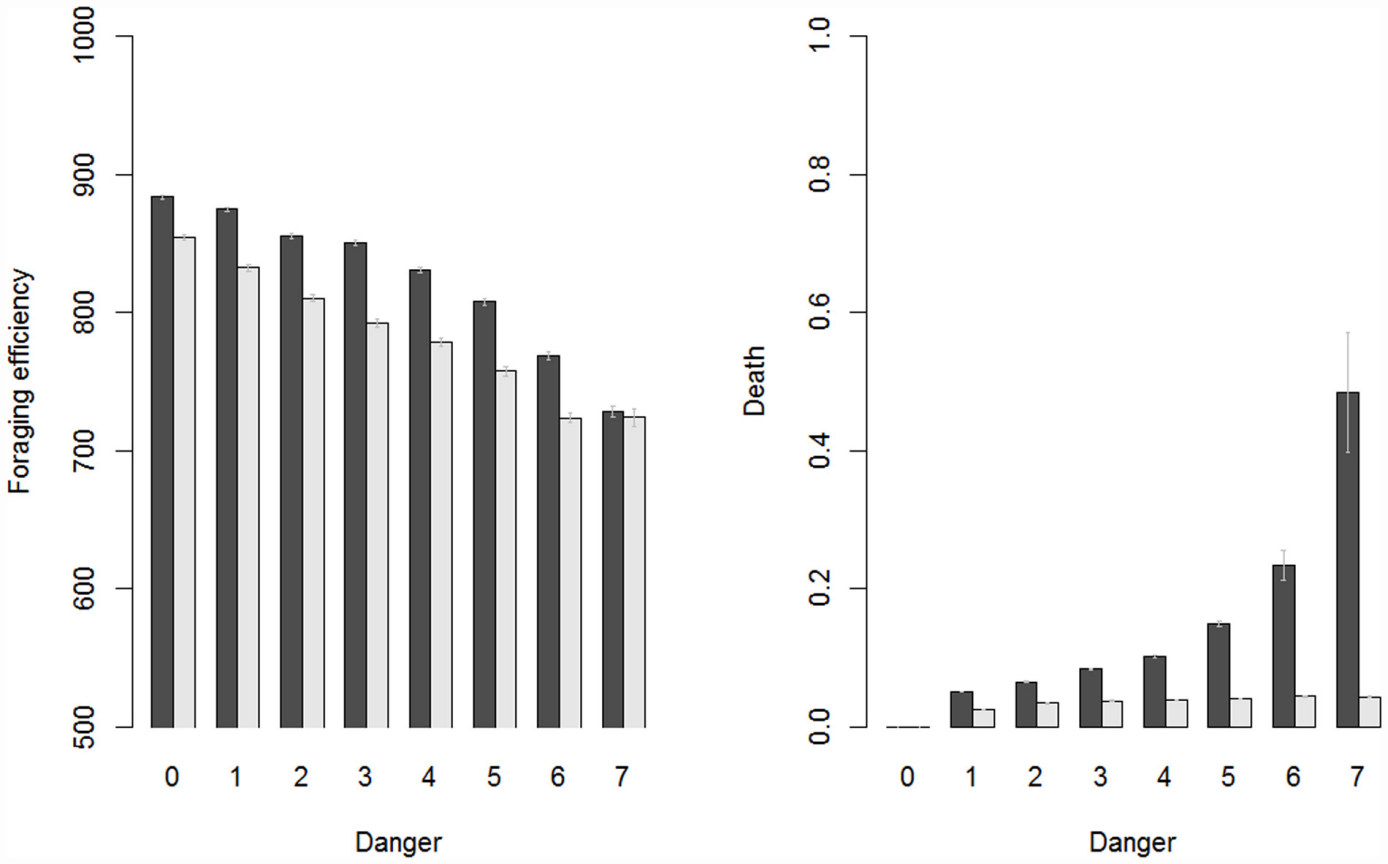
Tradeoff between foraging efficiency and safety. Black = no preparedness, gray = with preparedness. The means are derived from averaging time-step 25000–50000 for 100 repeats of each simulation. The standard errors are calculated across the 100 runs.

To further clarify this pattern or results, we directly explored the behavior in populations with- and without preparedness, and found that preparedness in general was detrimental for foraging efficiency by reducing the cumulative reward received (Fig 4, top). However, this reduction in foraging efficiency was associated with a sharply reduced risk of death (e.g., predation risk) from choosing a dangerous option (Fig 4, bottom).

This pattern was very similar across the different values of *P*_*change*_, which therefore are averaged for simplicity. Preparedness thus lead to a trade-off between foraging efficiency (due to decreased probability of sampling different options if the rewards are stochastic, or fixation on certain, possibly sub-optimal options) for an on average longer life-span. Moreover, this indicate that the evolution of negative preparedness, even when the environment is safe (*Danger* = 0, see Fig 3, right), is likely to depend on a drift-like process, which influence on foraging efficiency was small and attenuated by learning (Fig 4, top). The influence of preparedness on adaptive behavior in our model was thus beneficial in terms of survival probability in dangerous environments but detrimental in terms of foraging efficiency, suggesting that the preparedness mechanism in itself provides a constraint on the evolution of adaptive behavior [8,9,62]

### 3.3 Foraging efficiency of pure strategy populations with and without preparedness

**Fig 5:**
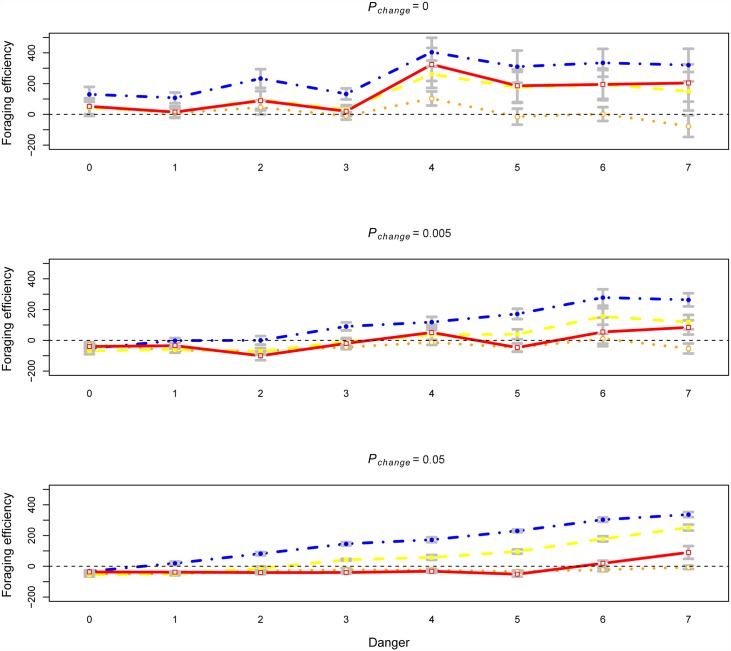
The differential foraging efficiency of pure learning strategies as a function of danger. Positive differential foraging efficiency indicates that foraging efficiency was greater with than without preparedness. Blue = Asocial learning (*O*_*0*_,*T*_*0*_), yellow = observational learning (*O*_*1*_,*T*_*0*_), red = parental learning (*O*_*0*_,*T*_*1*_), orange = advanced social learning (*O*_*1*_,*T*_*1*_). The means are derived from averaging time-step 25000–50000 for 10 repeats of each simulation. The standard errors are calculated across the 10 runs.

To further clarify how preparedness affects the behavior of the different social learning strategies, we next investigated the foraging efficiency (i.e., cumulative reward, Eq 1) of pure strategy populations, i.e., populations composed of agents with only one, fixed, learning strategy. Fig 5 shows the differential foraging efficiency of pure strategy populations as a function of preparedness, where a positive value indicates higher foraging efficiency with preparedness. This simulation revealed two main findings: (1) when the environment was stochastic (*P*_*change*_ > 0), preparedness generally had a detrimental effect on foraging efficiency when *Danger* was low, and (2) this pattern differed considerably for the different learning strategies.

For asocial learners, preparedness was generally beneficial, except when the environment was stochastic, but not dangerous. This pattern intuitively results from the same tradeoff as described above (c.f., Fig 4); preparedness lead to reduced foraging efficiency by producing behavioral fixation on a subset of, possibly sub-optimal, actions when the environment was changing. However, when the environment also was dangerous, the increased life-span resulting from preparedness outweighed this effect, as preparedness was the only way asocial learners can acquire a negative value for the dangerous options. This can be seen as a cost-benefit trade-off between behavioral flexibility and safety. Similarly, preparedness was generally beneficial for agents with the observational learning phenotype. Without preparedness, the phenotype can acquire both positive and negative representations by observing the actions of others but lack a representation of the value of the different actions at birth and thereby risk choosing a fatal outcome. This beneficial effect was qualified by how the environment was changing; in environments changing slowly (*P*_*change*_ = 0.005), preparedness reduced foraging efficiency for the pure observational conditioning strategy in all but the highest level of *Danger*.

In contrast, preparedness had a generally negative impact on foraging efficiency for the learning strategies with the parental learning allele (*T*_1_) when the environment was stochastic and not extremely dangerous. The reduced foraging efficiency was most pronounced for advanced social learning (Fig 5). Because the advanced social learning strategy was strongly represented across in our mixed strategy simulations (Fig 1), this resulted in the net reduction in foraging efficiency for populations with preparedness we described above (Fig 4). It is however important to note that the reduction in risk of death still promotes the evolution of preparedness. The difference between advanced social learning and the pure parental learning strategy can be illuminated by considering the differences in representational capacity of these strategies. Agents with the pure parental learning phenotype had a representation of the environment at birth (by vertical transmission), but only of the options with positive value in the ancestral generations. Because agents died when exposed to a fatal outcome, the pure parental learning strategy had no means to transmit information about the dangerous options vertically. Thus, when the environment is stable and/or highly dangerous, the benefit of preparedness outweighs the cost of the associated behavioral inflexibility. In contrast, advanced social learners could learn about the dangerous options both through horizontal observational learning, and transmit this information to their offspring by vertical transmission, which together allowed both behavioral precision and flexibility. Moreover, this suggests an explanation to why advanced social learning did not consistently outcompete asocial learning in the presence of preparedness when the environment was stable (Fig 1, bottom left). Advanced social learning requires the evolution of both observation (for representations of dangerous options) and parental learning (for vertical transmission of the dangerous options), neither which is strongly selected for in itself if the environment is stable or highly dangerous. In summary, the results of the pure strategy simulations suggest antagonistic effects between preparedness and social learning for foraging efficiency, in a way not directly anticipated by previous empirical findings [31,32].

### 3.4 Evolutionary novel dangers

So far, all the results pertain to situations where the same options were dangerous through the simulation, which corresponds to environments where the same predators, edibles, or actions constitute recurrent threats across evolutionary time. To explore the evolution of preparedness and social learning in environments where the dangerous options change, so that previously dangerous options become safe and previously safe options become dangerous (e.g., when a new predator arrives at a previously safe watering hole, or when social groups or coalitions shift), we ran simulations where the dangerous option changed with probability P_newdanger_ (set to 0.001).

**Fig 6:**
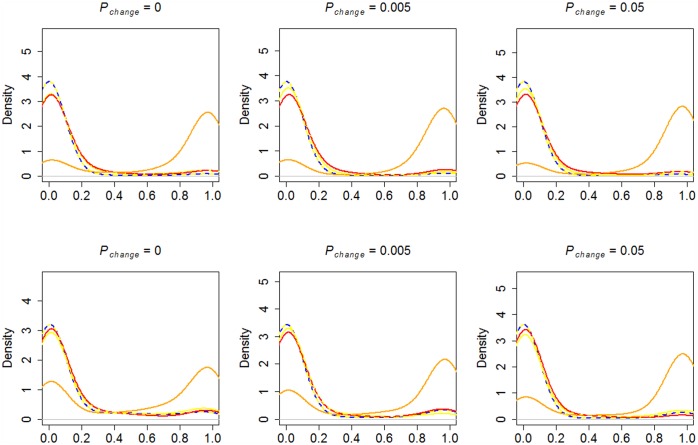
Phenotypic frequency densities with evolutionary novel dangers. Without (top) and with (bottom) preparedness. Density plot for the phenotypic frequency at the end-point (time step 50000) for the different strategies over 10 simulation runs. Solid lines: Danger > 0. Dotted lines: Danger = 0. Blue = Asocial learning (*O*_*0*_,*T*_*0*_), yellow = observational learning (*O*_*1*_,*T*_*0*_), red = parental learning (*O*_*0*_,*T*_*1*_), orange = advanced social learning (*O*_*1*_,*T*_*1*_).

Fig 6 shows a clear dominance of the advanced social learning phenotype, both in the presence (left) and absence (right) of preparedness, across different levels of reward stochasticity (in these simulations, *Danger* = 1. Higher values led to simulation failures due to population collapses). These results stand in contrast to the phenotypic frequencies in environments where the dangerous options are constant (Fig 1), and indicate that social learning is, intuitively, especially adaptive in environments where new dangers can emerge. It is important to note that the dominance of the advanced social learning over pure observational learning indicates that a representation of the dangerous options at birth is advantageous (provided P_newdanger_ is low enough for the acquired representations of the ancestral generation to still be on average valid), in line with the results for fixed dangers (Fig 1).

## 4. Discussion

In the present study, we asked how two contrasting cost-reducing adaptions, genetic preparedness and social learning, co-evolve and interact in dangerous environments. First, we examined if both preparedness and social learning typically evolve in dangerous environments, and found that this indeed was the case. The reason for this joint evolution was that these adaptations provided complimentary benefits: our simulations show that genetic preparedness generally reduce foraging efficiency, but result in a higher rate of survival in dangerous environments, thus reflecting a trade-off between behavioral flexibility and safety. Social learning, especially when combining mechanisms for both vertical and horizontal information transmission, generally came to dominate the population, especially when the environment was stochastic. However, even in this case, genetic preparedness reliably evolved. Thus, genetic preparedness affected the adaptiveness of social learning for avoiding dangerous outcomes in a manner that can be described as globally beneficial (by promoting survival) but locally detrimental (by reducing behavioral flexibility and thus foraging efficiency) [63]. In other words, the interaction between preparedness and social learning was both synergistic and antagonistic. Moreover, our simulations show that the relationship between genetic preparedness and social learning in dangerous environments is likely to be more complicated than suggested by previous empirical studies [31,32,37]. Studies of social learning in dangerous environments, commonly summarized under the “copy when costly” guise, which typically disregard genetic preparedness [64], might therefore benefit from attending to preparedness effects of the type we have simulated in the present study, given their known importance for individual learning and decision making in human and non-human animal behavior [16,26,42,45].

The effect of genetic preparedness on social learning in our simulations can be viewed as a decision making *bias*, similar to other evolutionary derived biases based on “better safe than sorry” trade-offs. For example, many animals exhibit learning and decision making biases in interaction with possible predators [1]. These biases serve to promote fitness by minimizing the possible costs of predation, by sacrificing accuracy or expected payoffs [1,65,66]. Such biases are often thought to promote adaptive behavior by providing robustness, formally defined as the insensitivity of the behavioral systems to environmental perturbations [26]. Our results closely align with this perspective, by showing that genetic preparedness reduce foraging efficacy and behavioral flexibility, but promote survival across a variety of environments.

The existence of this behavioral bias in our simulation results correspond to empirical human decision making biases involving prepared stimuli, such as spiders, snakes, and threatening human faces [43,67,68]. For example, the mere presentation of snake images as decision cues led to corrupted decision making when snakes were unreliable predictors of danger (mild electric shocks) [43]. Our model predict that such biases would generalize to situations involving social learning, which is easily testable in humans or non-human primates, and possibly also in other species. Furthermore, it is possible that the combination of preparedness and social learning, rather than individual learning, might underpin widespread fears and phobias in humans, towards for example snakes, as direct learning experiences are relatively rare in the etiology of phobias [36,69,70].

In environments where new dangers could emerge, advanced social learning was dramatically dominant, whereas preparedness, intuitively, made little contribution to adaptive behavior (Fig 6). It is possible that advanced social learning, which arguably is employed by humans [10], is the foremost mechanism to avoid dangers in the modern world (e.g. without the combination of parental learning and observational learning, crossing a highly trafficked city street would be lethal). It is telling that modern, highly lethal dangers, such as cars or electric outlets, seldom are subject of phobias in the way evolutionarily old dangers, such as snakes or spiders, are [15–17]. Generally, our results also showed that vertical, rather than horizontal, transmission was the most important ingredient for the adaptiveness of social learning, but, importantly, that vertical and horizontal learning are beneficially interacting as expressed in the success of the advanced social learning strategy.

In contrast to some recent models of social learning based on the producer-game in which scrounger individuals can benefit from the rewards uncovered by producer individuals, [71,72], the fitness of the learning strategies in our model was not frequency dependent. Instead, any benefit of social learning arose as a direct consequences of *experiential* sampling, i.e., the individual agent’s decisions to sample certain actions and avoiding others. This approach has been used in previous simulation studies based on multi-armed bandits [46], and is furthermore supported by the known importance of the interaction of social information and experiential sampling in humans [73–75]. However, extending the present findings to a frequency-dependent context where the number of agents exploiting a resource influences its value, represent an important venue for future modeling work. In contrast to previous, typically analytical, models of social learning, that have studied the effect of environmental change on the evolution of social learning, and found that a high rate of change favors individual learning [49,50], our model showed that horizontal social transmission can be beneficial under such circumstances. The likely reason for this dissimilarity stem from our assumption that agents simultaneously can learn from both individual and social outcomes, which is grounded in the human learning literature (e.g., [54,56]).

In line with the recently emerging perspective that evolutionary modeling needs to explicitly model the *mechanisms* that produce behavior, rather than agnostically focusing on fitness differentials (i.e., the phenotypic gambit) [8,9,62,76], our results show how explicit mechanisms interact to promote adaptive behavior. However, this focus on behavioral mechanisms raises an important question: are we modeling the *right* mechanisms? Our model of individual learning and decision making is directly based on research in psychology and neuroscience, which have showed that the Rescorla-Wagner learning rule, along with other closely related rules, provides a concise description of both behavioral and neural aspects of learning from rewards and punishment [52,77,78], as well as being evolutionarily robust [60]. The implementation of how preparedness and learning interacts (as competing influences at the decision stage) is based on behavioral experiments conducted in our lab [43], but other, similar, implementations are possible [45,79,80]. The implementation of observational learning (*O*_1_) was identical to individual learning, and is supported by previous behavioral [55] and neural [57] findings in humans. Moreover, this implementation is directly in line with the emerging view that social learning rests on the same learning mechanisms as individual learning, rather than specialized cognitive mechanisms[40,41,81]. The present model of observational learning only assume that the can derive information from the behavior of others (i.e., has a non-zero learning rate), and that other′s rewards and punishments have a vicarious value. Both assumptions are supported by a wide range of studies [55,63,70,82–84]. Empirically, these attentional and reinforcement aspects of social learning can emerge either from individual learning, such as second-order conditioning [85], evolutionary adaptions [41], or any combination thereof.

Finally, the implementation of parental learning (*T*_1_) in our model was highly abstract (e.g., a direct transmission of the parent’s action values to the offspring at the time of conception) in order to keep the reproduction stage (i.e., asexual and haploid) as simple as possible. A more realistic implementation might involve a period where the offspring is co-residing with the parent agent, with learning from the parent being a gradual process unfolding over time. Development of such more complex models is outside the scope of the present study, but is an important topic for future developments of our model.

In summary, the present simulation-based study uncovered a number of theoretically novel interactions between genetic preparedness and social learning. Our results show that both genetic preparedness and social learning are likely to evolve in dangerous environments. Furthermore, our findings show that genetic preparedness is likely to lead to a trade-off between foraging efficiency and survival, also in populations of social learners. Studies of both individual and social learning might therefore benefit from considering the evolutionary environment of the species in questions, also if the species is human, to understand behaviors that are seemingly suboptimal, but might reflect adaptive tradeoffs between flexibility and safety [62].

## Supporting Information

S1 Code Computer code to run the basic simulation (3.0–3.2). Can be opened with NetLogo (freely available from https://ccl.northwestern.edu/netlogo/).
(NLOGO)

S2 Code Computer code to run the pure strategy simulation (3.3). Can be opened with NetLogo (freely available from https://ccl.northwestern.edu/netlogo/).
(NLOGO)

S3 Code Computer code to run the changing danger simulation (3.4). Can be opened with NetLogo (freely available from https://ccl.northwestern.edu/netlogo/).
(NLOGO)

S1 Fig Stochastic equilibrium frequencies without (top) and with (bottom) preparedness. The plots depict the percentage of time each strategy was near fixation (defined as constituting at least 90% of the population). Blue = Asocial learning (*O*_*0*_,*T*_*0*_), yellow = observational learning (*O*_*1*_,*T*_*0*_), red = parental learning (*O*_*0*_,*T*_*1*_), orange = advanced social learning (*O*_*1*_,*T*_*1*_). The standard errors are calculated across the 100 runs.
(PDF)

S2 Fig Phenotypic end-point frequencies without (top) and with (bottom) preparedness. Blue = Asocial learning (*O*_*0*_,*T*_*0*_), yellow = observational learning (*O*_*1*_,*T*_*0*_), red = parental learning (*O*_*0*_,*T*_*1*_), orange = advanced social learning (*O*_*1*_,*T*_*1*_). The means are derived from the end-point distribution (time step 50000) averaged over 100 runs of each simulation. The standard errors are calculated across the 100 runs.
(TIFF)
