# Supplementary figures and images for: Co-Evolution of Social Learning and Evolutionary Preparedness in Dangerous Environments
Source: PLoS One. 2016 Aug 3;11(8):e0160245. doi: 10.1371/journal.pone.0160245 (PMC4972391; doi:10.1371/journal.pone.0160245)

$P_{change} = 0$ 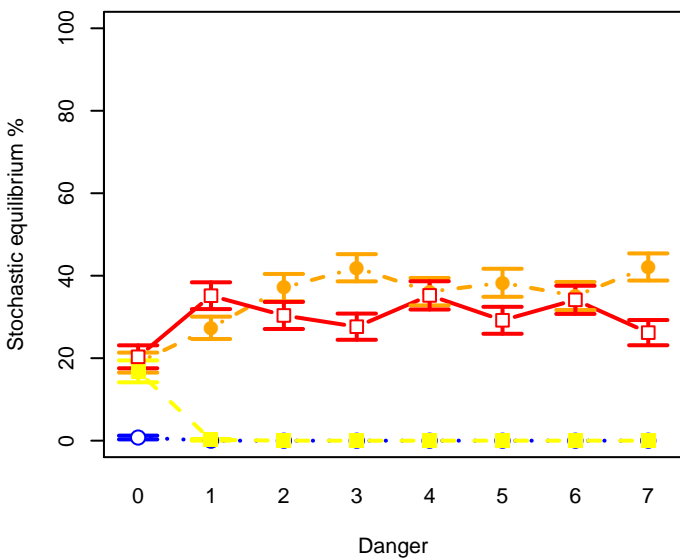 $P_{change} = 0.005$ 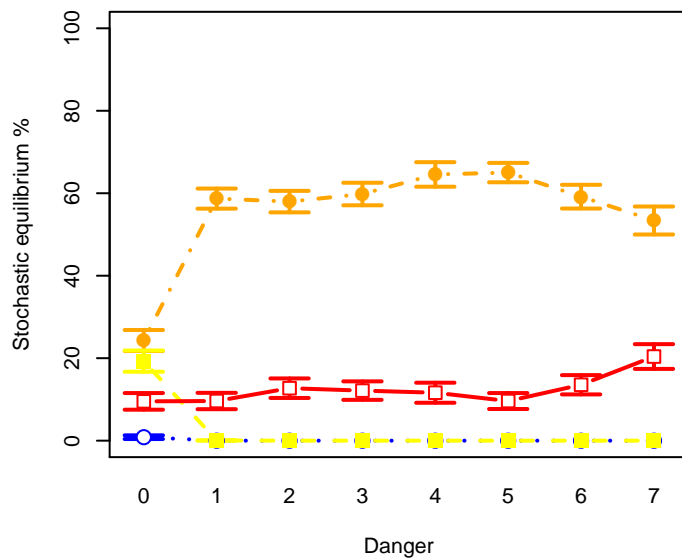 $P_{change} = 0.05$ 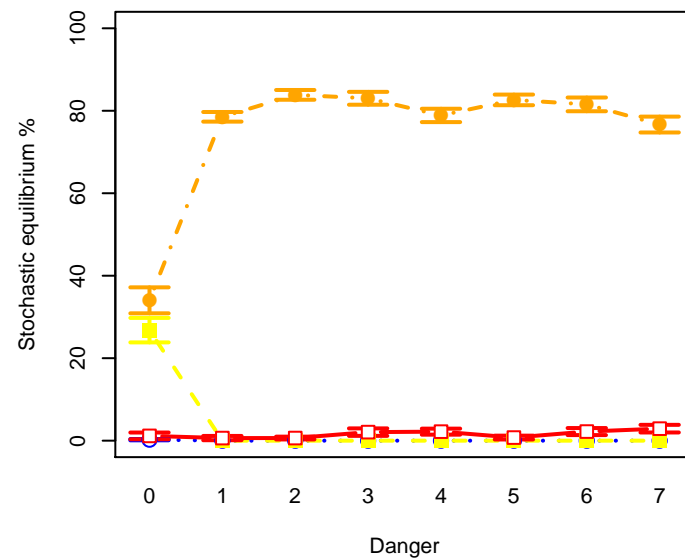 $P_{change} = 0$ 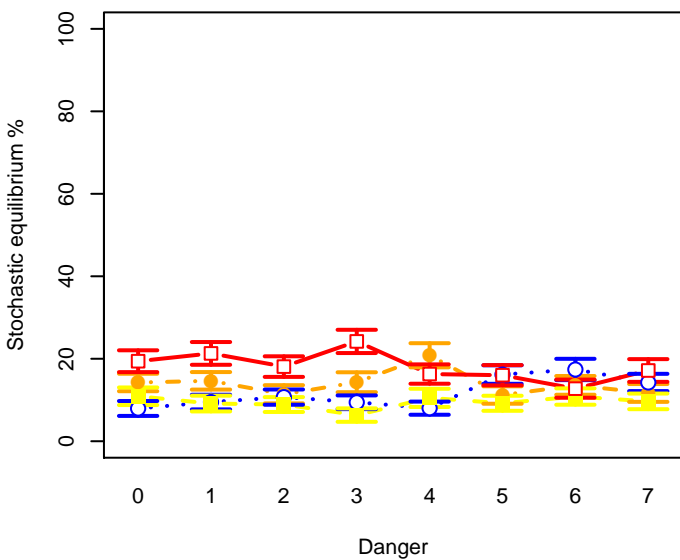 $P_{change} = 0.005$ 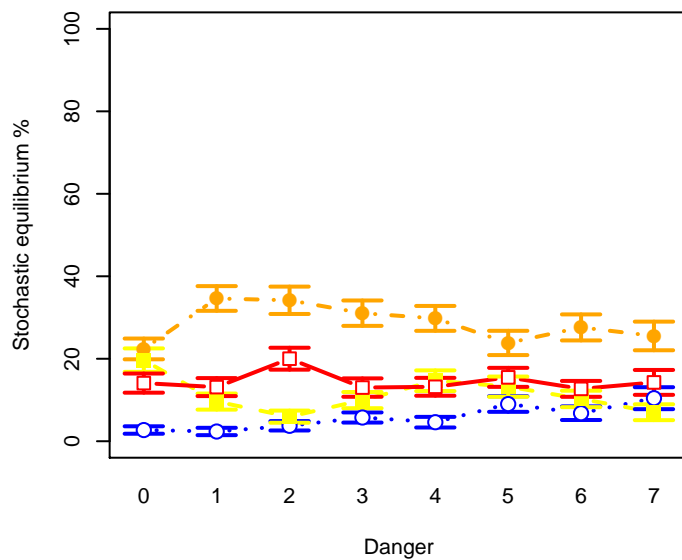 $P_{change} = 0.05$ 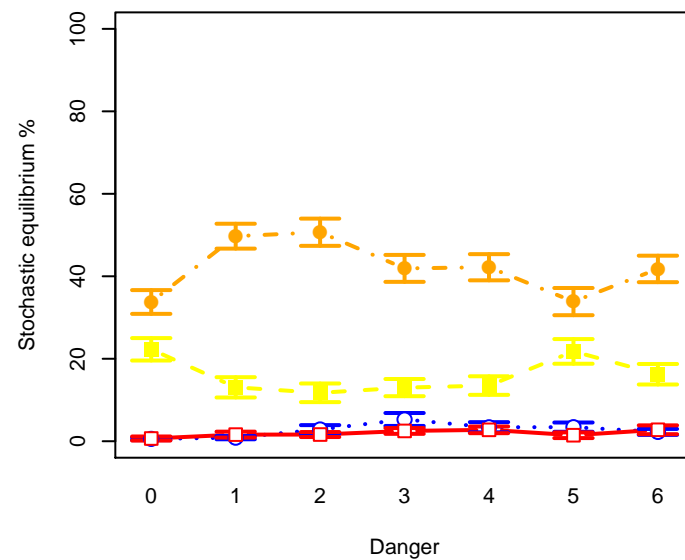

Supplement: S1 Fig — The plots depict the percentage of time each strategy was near fixation (defined as constituting at least 90% of the population). Blue = Asocial learning (O0,T0), yellow = observational learning (O1,T0), red = parental learning (O0,T1), orange = advanced social learning (O1,T1). The standard errors are calculated across the 100 runs. (PDF) [file pone.0160245.s004.pdf]

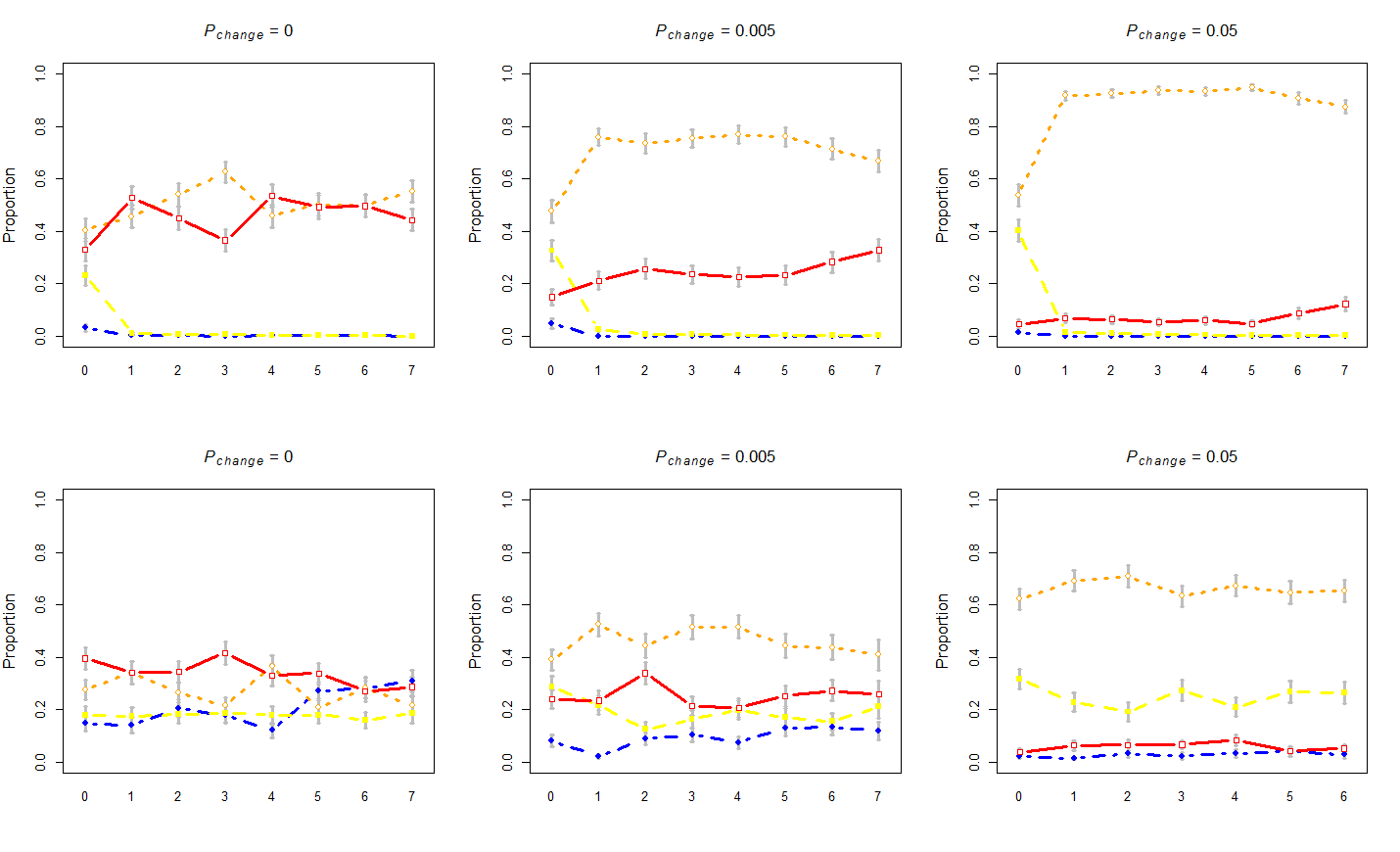

Supplement: S2 Fig — Blue = Asocial learning (O0,T0), yellow = observational learning (O1,T0), red = parental learning (O0,T1), orange = advanced social learning (O1,T1). The means are derived from the end-point distribution (time step 50000) averaged over 100 runs of each simulation. The standard errors are calculated across the 100 runs. (TIFF) [file pone.0160245.s005.tiff]
